# Factors of post-vaccination serological testing coverage among hepatitis B-exposed children in Fujian, China: a population-based cohort study (2021–2023)

**DOI:** 10.3389/fpubh.2026.1797570

**Published:** 2026-04-09

**Authors:** Xinxin Huang, Yongfan Chen, GuiHua Liu, Chengyu Huang, Xiaomeng Xue, Wenzhao Lin, Xiumin Jiang

**Affiliations:** 1 Fujian Maternity and Child Health Hospital College of Clinical Medicine for Obstetrics & Gynecology and Pediatrics, Fujian Medical University, Fuzhou, China; 2 School of Nursing, Fujian Medical University, Fuzhou, China

**Keywords:** cohort study, hepatitis B virus, mother-to-child transmission, post-vaccination serological testing, risk factors

## Abstract

**Background:**

Mother-to-child transmission (MTCT) of the hepatitis B virus (HBV) is still a major public health concern worldwide, especially in high-burden nations like China. In order to assess the effectiveness of children vaccinations and stop MTCT, post-vaccination serological testing (PVST) is crucial. However, there are obstacles to PVST implementation.

**Objective:**

The purpose of study is to evaluate the existing local strategies for preventing MTCT and suggest possible improvements by analyzing the dynamics of PVST coverage in children exposed to HBV and the factors that influence it in Fujian Province between 2021 and 2023.

**Methods:**

Data from the China Immunization Planning Information System and the China Information System for Prevention of Mother-to-Child Transmission was used in the population-based cohort research. To describe the current status of PVST implementation and find factors linked to it, multivariable logistic regression were used, stratified by high-risk (maternal HBV DNA ≥2 × 10^5^ IU/ml or HBeAg-positive) and common HBV-exposed children.

**Results:**

From 32.4% in 2021 to 79.2% in 2023, PVST coverage rose dramatically, with higher rates among high-risk children (70.5%) than among children with common exposure (53.2%). Failure rates for MTCT dropped from 0.8 to 0.3%. However, the recommended 1–2-month range was exceeded, with over half of children undergoing PVST more than 2 months following immunization. For children with high exposure to HBV, antiviral medication use (OR = 1.575, 95% *CI* = 1.452–1.708, *P* < 0.01) and pregnancy duration ≥37 weeks (OR = 1.433, 95% *CI* = 1.198–1.708, *P* < 0.01) were revealed to be risk factors for not completing PVST. For children with common exposure to HBV, one factor for not completing PVST was the mother's employment as a labor or farmer (OR = 0.833, 95% *CI* = 0.780–0.889, *P* < 0.01). For all children exposed to HBV, maternal age ≥30 years, education ≤ high school, and maternal parity ≥2 times were important factors for non-completion.

**Conclusions:**

In terms of PVST coverage and MTCT control, Fujian Province made impressive strides. Maintaining the progress toward MTCT elimination requires the antepartum and postpartum observation periods incorporate a consistent instructional procedure.

## Background

The hepatitis B virus (HBV) is vertically transmitted by mother-to-child transmission (MTCT), sexual contact, and blood. HBV infection can lead to acute and chronic infection characterized by hepatocellular damage (1). In 15%−40% of cases, chronic HBV infection leads to cirrhosis or hepatocellular carcinoma, and it is the primary cause of liver disease-related mortality globally (2). According to the Global Hepatitis Report 2022, the global prevalence of HBV infection is approximately 257 million (3.2%), with 1.8 million (1.4%) of those infections occurring in children under 5 years of age (3). In response to this pervasive public health crisis, the World Health Organization (WHO) Western Pacific Region has recommended the elimination of MTCT of HBV as a priority strategy, with a clear requirement to achieve an HBsAg positivity rate of <0.1% in children under 5 years of age by 2030 (4).

China is among the countries with the highest burden of HBV-related diseases (5), and MTCT is the most significant route of chronic HBV infection in the country (6). Consequently, interrupting MTCT is a priority for the prevention and control of HBV in China. By implementing comprehensive prevention and control strategies, such as the universal hepatitis B vaccination program, passive immunization with hepatitis B immunoglobulin, and antiviral treatment for high-risk pregnant women, China has made significant progress in the prevention and management of HBV MTCT (7). As a result, between 2015 and 2020, the HBV infection rate among pregnant women decreased by an average of 5.27% per year, for a total decrease of 25.44% (8). However, between 700,000 and 1,000,000 HBsAg-positive mothers still give birth in China each year, with about one-third of them being HBeAg-positive (9). It is concerning that children born to pregnant women who are HBeAg-positive or have a high viral load (HBV DNA ≥2 × 10^5^ IU/ml) still have a 3%−10% chance of MTCT even if they receive hepatitis B vaccination in combination with HBV immunoglobulin (10–13). Approximately 50,000 infants in China contract HBV each year, and, if prompt action is not taken, 80%−90% of these infants will develop chronic HBV carriage, with a high probability of severe health sequelae (14, 15).

In this context, post-vaccination serological testing (PVST) of children exposed to HBV is of significant clinical value. PVST not only provides an objective criterion for assessing the efficacy of mother-to-child hepatitis B interruption, but it is also a crucial intervention period for administering prompt remedial vaccination to children with insufficient immune response (16, 17). Nevertheless, China has not yet integrated PVST for children exposed to HBV into the country's routine testing system. Only a pilot program has conducted small-scale sampling, with notable regional variations in coverage. For example, the Tongzhou District of Beijing had a PVST rate of 85.88%, but the urban, rural, and pastoral sections of Qinghai Province had corresponding PVST rates of 14.17%, 12.31%, and 8.06% (18, 19). This discrepancy highlights the need for expanding the PVST good practice standard to areas with low PVST rates, with a view to enhancing HBV prevention.

Multiple PVST pilot programs are being gradually implemented in China, and the clinical feasibility and necessity have been supported by evidence-based data (20). Fujian Province, a region with a medium to high prevalence of HBV, serves as a pivotal area for HBV prevention and control. The maternal HBV infection rate in this province can reach up to 11.39%, underscoring the significance of maternal health in this context (21). In recent years, Fujian Province has worked hard to enhance PVST implementation through follow-up interventions, information and data integration, and policy assistance. The objective of this study is to conduct a comprehensive analysis of the dynamic changes in PVST coverage and its associated factors in Fujian Province from 2021 to 2023. This analysis aims to provide a direct reference for the optimization of strategies in endemic areas of a similar level in China. Additionally, the findings from this study can contribute to the development of effective prevention and control programs in mid-to-high endemic areas worldwide.

## Methods

### Participants

The target population for this study comprised infants born to HBsAg-positive mothers between January 1, 2021, and December 31, 2023, in Fujian Province.

### Specimen collection procedures

In this cohort study, children exposed to HBV were followed up by examining the China Immunization Planning Information System and the China Information System for Prevention of Mother-to-Child Transmission (PMTCT) of HIV, Syphilis, and Hepatitis B. Basic data on mothers and infants were gathered, together with data on children's immunization status, follow-up, and PVST implementation.

The judgment criteria are as follows: (1) normal response to immunization: HBsAg negative and anti-HBs positive; (2)low response to immunization: HBsAg negative and anti-HBs weakly positive; (3) no response to immunization: both HBsAg and anti-HBs negative; (4) failure of immunization and occurrence of MTCT: HBsAg positive, with or without HBeAg positive; (5) high-exposure risk children are defined as born to mothers with an HBV-DNA test result of ≥2 × 10^5^ IU/ml. If the mother's DNA was not tested during pregnancy, high-exposure risk children are defined as born to mothers with HBeAg-positive. The rest are classified as common exposure risk children.

### Data analysis

The database was obtained from the China PMTCT of HIV, Syphilis, and Hepatitis B Information System. Cases of duplicate records (duplicate reporting of the same follow-up subject), omissions (e.g., twin births but only one follow-up card was filled out), and missing key information (e.g., incomplete PVST results) were verified multiple times until consistency comparisons were matched. The statistical analysis was conducted using R 4.4.2 and SPSS 27.0. The categorical data was expressed as frequency and constituent ratio (*n*%), and the comparison between groups was made using chi-square test or Fisher's exact probability method. All variables with a *P*-value less than 0.05 were then included in the multivariable logistic regression model. The results were visualized using a forest plot to display odds ratios (ORs) and 95% confidence intervals (CIs). *P*-value <0.05was deemed statistically significant in the two-sided test.

## Result

### PVST information

**Figure 1:**
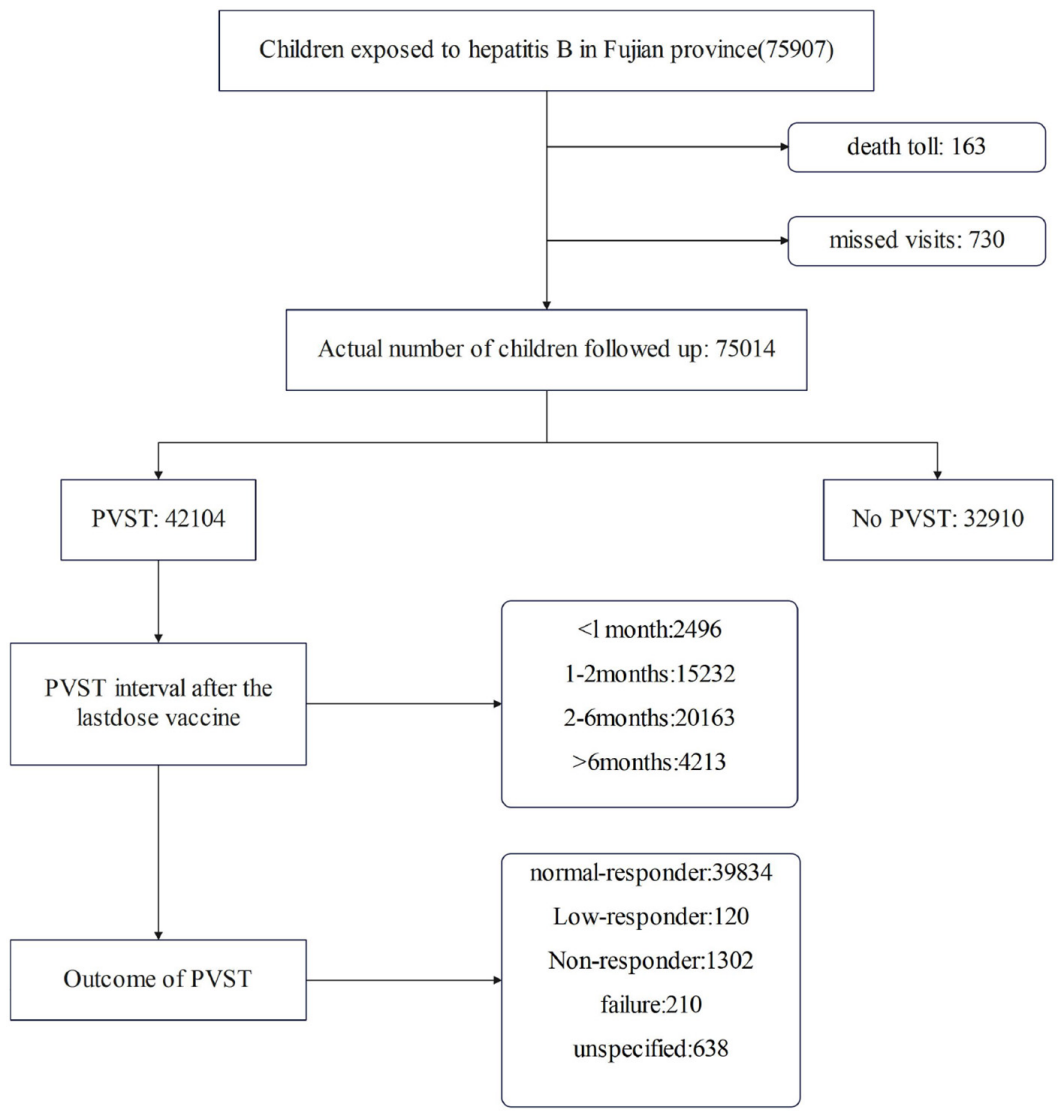
PVST flow chart in Fujian Province, China.

A total of 75,907 children who were born to HBsAg-positive mothers in Fujian Province between January 1, 2021, and December 31, 2023, were included in the study. 75,014 of them finished the completed follow-up. Of these, 42,104 (56.1%) children had PVST after receiving full hepatitis B immunization, and the PVST coverage increased from 32.4% in 2021 to 79.2% in 2023. The PVST coverage for high-risk exposed children was 70.5%, which was higher than the PVST coverage for commonly exposed children (53.2%). The results of PVST showed that the HBV mother-to-child blockade failure rate dropped from 0.8% to 0.3%; however, over half of the children experienced a gap of more than 2 months between their last vaccination and PVST. Please consult Figure 1 and Table 1 for summary results.

**Table 1:** Follow-up table for children exposed to HBV (*n* = 75,907).

Follow-up and immunological findings		2021	2022	2023	Total
Total		30,405	23,942	21,560	75,907
Death toll		54	62	47	163
Missed visits		321	318	91	730
Actual number		30,030	23,562	21,422	75,014
Status of PVST coverage					
No		20,305 (67.6)	8,157 (34.6)	4,448 (20.8)	32,910 (44.9)
Yes		9,725 (32.4)	15,405 (65.4)	16,974 (79.2)	42,104 (56.1)
PVST interval (month) after the last dose vaccine					
<1		541 (5.6)	1005 (6.5)	950 (5.6)	2496 (5.9)
1–2		3,242 (33.3)	5,155 (33.5)	6,835 (40.3)	15,232 (36.2)
2–6		4,816 (49.5)	7,379 (47.9)	7,968 (46.9)	20,163 (47.9)
>6		1,126 (11.6)	1,866 (12.1)	1,221 (7.2)	4,213 (10.0)
Outcome of PVST					
Normal-responder		8,921 (91.7)	14,663 (95.2)	16,250 (95.7)	39,834 (94.6)
Low-responder		34 (0.3)	42 (0.3)	44 (0.3)	120 (0.3)
Non-responder		271 (2.8)	501 (3.3)	530 (3.1)	1,302 (3.1)
Failure		79 (0.8)	73 (0.5)	58 (0.3)	210 (0.5)
Unspecified		420 (4.3)	126 (0.8)	92 (0.5)	638 (1.5)
PVST coverage of highly or commonly exposed children					
Highly exposed children	Total	5,587	3,603	3,509	12,699
	PVST completed	3,260 (53.8)	2,759 (76.5)	2,930 (83.5)	8,949 (70.5)
	PVST not completed	2,327 (47.2)	844 (23.5)	579 (16.5)	3,750 (29.5)
Commonly exposed children	Total	24,443	19,959	17,913	62,315
	PVST completed	6,465 (26.5)	12,646 (63.3)	14,044 (78.4)	33,155 (53.2)
	PVST not completed	17,978 (73.5)	7,313 (36.7)	3,869 (21.5)	29,160 (46.8)

### Characteristics of follow-up and lost-to-follow-up groups

There were significant differences between the two groups in most demographic and clinical characteristics (*P* < 0.05), except for maternal age (*P* = 0.082) and the mode of delivery (*P* = 0.136). Specifically, compared with the follow-up group, the lost-to-follow-up group had a higher proportion of individuals from ethnic minorities, those with lower educational levels (junior middle school and below), and those whose occupation was recorded as “at home.” A comparison of baseline characteristics between the followed-up and lost-to-follow-up groups is presented in Table 2.

**Table 2:** Characteristics of follow-up and lost-to-follow-up groups (*n* = 75,744).

Variables	Followed up (*n* = 75,014)	Lost-to follow-up (*n* = 730)	χ^2^	*P*
**Maternal age (years)**			5.002	0.082
<30	23,156 (30.9)	253 (34.7)		
30–34	33,805 (45.1)	315 (43.2)		
≥35	18,053 (24.1)	162 (22.2)		
**Ethnic group**			161.219	**<0.001**
Han Chinese ethnic group	73,538 (98.0)	667 (91.4)		
Minority	1,476 (2.0)	63 (8.6)		
Maternal education, *n* (%)			33.843	**<0.001**
Bachelor degree and above	32,293 (43.1)	264 (36.2)		
High school	18,548 (24.7)	157 (21.5)		
Junior middle school and below	24,173 (32.2)	309 (42.3)		
**Maternal occupation**			117.030	**<0.001**
At home	40,558 (54.1)	511 (70.0)		
Management, professional and technical personnel	10,139 (13.5)	32 (4.4)		
Business, service, freelance	5,435 (7.3)	75 (10.3)		
Workers and farmers	5,214 (7.0)	24 (3.3)		
Others	13,668 (18.2)	88 (12.1)		
**Marriages**			18.953	**<0.001**
Married	73,595 (98.1)	700 (95.9)		
Others	1,419 (1.9)	30 (4.1)		
**Maternal parity (times)**			28.940	**<0.001**
1	42,122 (56.2)	340 (46.6)		
2	23,741 (31.7)	270 (37.0)		
≥3	9,151 (12.2)	120 (16.4)		
**Antiviral medication use**			6.344	**0.012**
No	65,998 (88.0)	620 (84.9)		
Yes	9,016 (12.0)	110 (15.1)		
**Pregnancy duration**			35.778	**<0.001**
<37	4,174 (5.6)	78 (10.7)		
≥37	70,840 (94.4)	652 (89.3)		
**Mode of delivery**			2.218	0.136
Vaginal delivery	43,990 (58.6)	448 (61.4)		
Cesarean section	31,024 (41.4)	282 (38.6)		
**Places of delivery**			50.307	**<0.001**
Midwifery institutions at the county level and below	40,386 (53.8)	297 (40.7)		
Municipal and higher midwifery institutions	34,628 (46.2)	433 (59.3)		
**Gender of children**			0.232	0.630
Female	34,169 (45.6)	326 (44.7)		
Male	40,845 (54.5)	404 (55.3)		
**Birth weight of children (g)**			5.846	**0.016**
<2,500	3,852 (5.1)	52 (7.1)		
≥2,500	71,162 (94.9)	678 (92.9)		
**Exposure levels in children**			152.980	**<0.001**
Commonly exposed children	62,315 (83.1)	480 (65.8)		
Highly exposed children	12,699 (16.9)	250 (34.3)		

Bold values indicate statistical significance (*p* < 0.05).

### Basic information

From January 1, 2021, to December 31, 2023, a total of 75,014 children were born to mothers under the age of 35 (approximately 75%), with an educational attainment of a bachelor's degree or higher (approximately 46%), a higher proportion than the average of mothers currently residing at home, and 15.82% of mothers who received medicine treatment during pregnancy. Among the total of 75,014 children who had been exposed to HBV, 22,897 (54.4%) were male. Table 3 provides a more thorough analysis of these findings.

**Table 3:** PVST status for 2021–2023 (*n* = 75,014).

Variables	Total (*n* = 75,014)	PVST completed (*n* = 32,910)	PVST not completed (*n* = 42,104)
Maternal age (years)			
<30	23,156 (30.9)	7,388 (22.4)	15,768 (37.5)
30–34	33,805 (45.1)	16,183 (49.2)	17,622 (41.9)
≥35	18,053 (24.1)	9,339 (28.4)	8,714 (20.7)
Ethnic group			
Han Chinese ethnic group	73,538 (98.0)	32,250 (98.0)	41,288 (98.1)
Minority	1,476 (2.0)	660 (2.0)	816 (1.9)
Maternal education			
Bachelor degree and above	32,293 (43.0)	12,720 (38.7)	19,573 (46.5)
High school	18,548 (24.7)	8,740 (26.6)	9,808 (23.3)
Junior middle school and below	24,173 (32.2)	11,450 (34.8)	12,723 (30.2)
Maternal occupation			
At home	40,558 (54.1)	17,524 (53.2)	23,034 (54.7)
Management, professional and technical personnel	10,139 (13.5)	4,056 (12.3)	6,083 (14.4)
Maternal occupation			
Business, service, freelance	5,435 (7.2)	2,220 (6.7)	3,215 (7.6)
Laborers, farmers	5,214 (7.0)	2,521 (7.7)	2,693 (6.4)
Others	13,668 (18.2)	6,589 (20.0)	7,079 (16.8)
Marriages			
Married	73,595 (98.1)	32,252 (98.0)	41,343 (98.2)
Others	1,419 (1.9)	658 (2.0)	761 (1.8)
Maternal parity (times)			
1	42,122 (56.2)	16,717 (50.8)	25,405 (60.3)
2	23,741 (31.6)	11,524 (35.0)	12,217 (29.0)
≥3	9,151 (12.2)	4,669 (14.2)	4,482 (10.6)
Antiviral medication use			
No	65,998 (88.0)	30,558 (92.9)	35,440 (84.2)
Yes	9,016 (12.2)	2,352 (7.1)	6,664 (15.8)
Pregnancy duration (weeks)			
<37	4,174 (5.6)	2,049 (6.2)	2,125 (5.0)
≥37	70,840 (94.4)	30,861 (93.8)	39,979 (95.0)
Mode of delivery			
Vaginal delivery	43,990 (58.6)	19,014 (57.8)	24,976 (59.3)
Cesarean section	31,024 (41.4)	13,896 (42.2)	17,128 (40.7)
Places of delivery			
Midwifery institutions at the county level and below	40,386 (53.8)	17,573 (53.4)	22,813 (54.2)
Municipal and higher midwifery institutions	34,628 (46.2)	15,337 (46.6)	19,291 (45.8)
Gender of children			
Female	34,169 (45.6)	14,962 (45.5)	19,207 (45.6)
Male	40,845 (54.5)	17,948 (54.5)	22,897 (54.4)
Birth weight of children(g)			
<2500	3,852 (5.1)	1,801 (5.5)	2,051 (4.9)
≥2500	71,162 (94.9)	31,109 (94.5)	40,053 (95.1)

### Factors correlated with PVST

**Figure 2:**
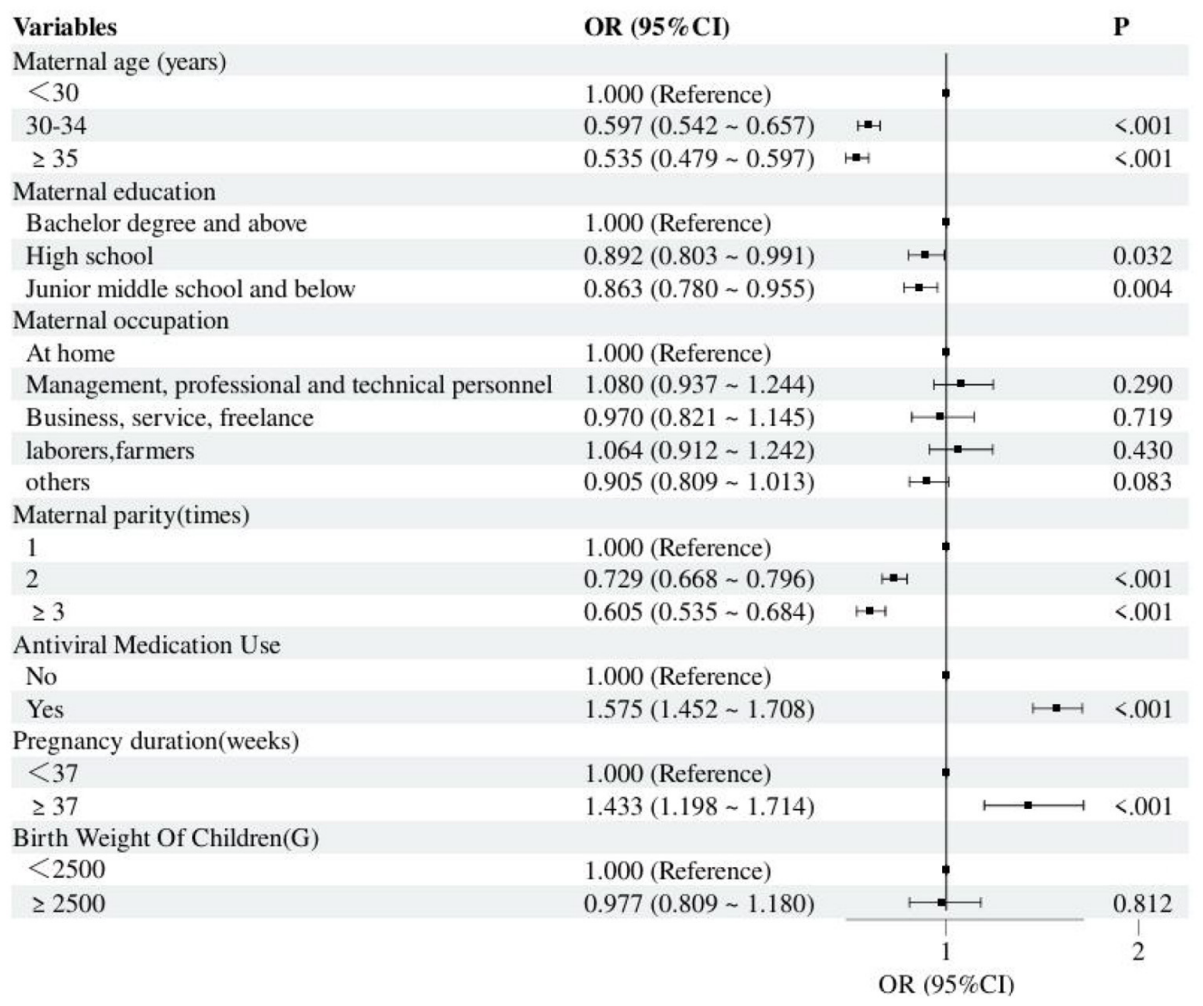
Multivariable logistic regression results for PVST not being completed of highly exposed children.

**Figure 3:**
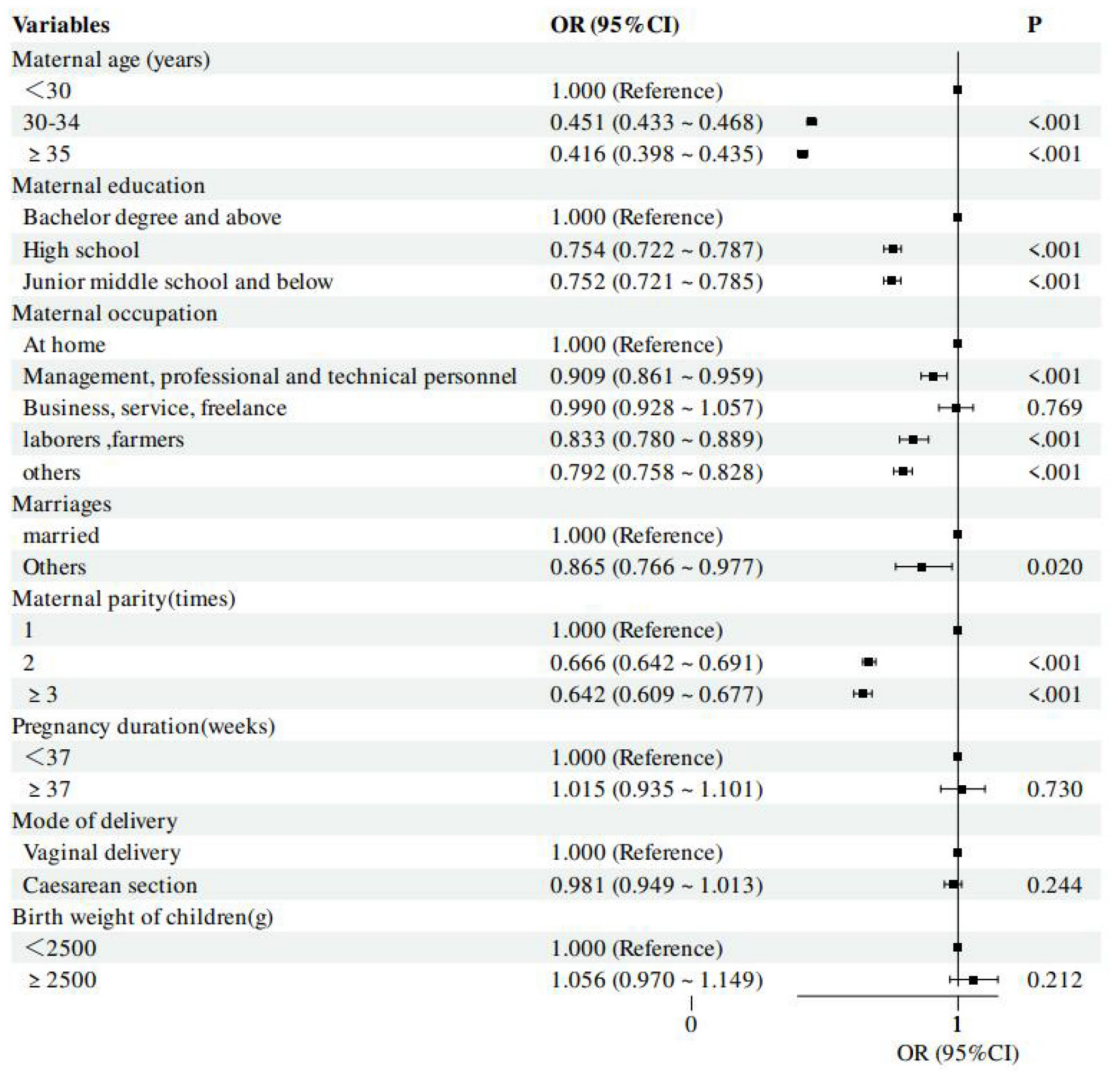
Multivariable logistic regression results for PVST not being completed of commonly exposed mothers.

The factors that were statistically significant in the univariate analysis were fitted using multivariable logistic regression: The factors affecting PVST vary among children with different risks of hepatitis B exposure. For children with a high risk of exposure to HBV, antiviral medication use (OR = 1.575, 95% *CI* = 1.452–1.708, *P* < 0.01) and pregnancy duration ≥37 (OR = 1.433, 95% *CI* = 1.198–1.714, *P* < 0.01)were found to be risk factors for not completing PVST. On the other hand, for children with common exposure to HBV, being a laborer-farmer (OR = 0.833, 95% *CI* = 0.780–0.889, *P* < 0.01) was found to be a factor for not completing PVST. For all hepatitis B-exposed children, the following risk factors were found to be associated with not completing PVST: maternal age ≥30 years, among 30–34years (for high exposure, OR = 0.597, 95% *CI* = 0.542–0.657, *P* < 0.01) (for common exposure, OR = 0.451, 95% *CI* = 0.433–0.468, *P* < 0.01); high school education (for high exposure, OR = 0.892, 95% *CI* = 0.803–0.991, *P* = 0.032) (for common exposure, OR = 0.754, 95% *CI* = 0.722–0.787, *P* < 0.01); and Maternal parity = 2 times (for high exposure, OR = 0.729, 95% *CI* = 0.668–0.796, *P* < 0.01) (for common exposure, OR = 0.666, 95% *CI* = 0.642–0.691, *P* < 0.01). Tables 4, 5, and Figures 2, 3 present the results in detail.

**Table 4:** Risk factors associated with PVST not being completed of highly exposed children (*n* = 12,699).

Variables	Total (*n* = 12,699)	No PVST (*n* = 3,750)	PVST (*n* = 8,949)	χ^2^	*P*
**Maternal age (years)**				180.309	**<0.001**
<30	3,936 (31.0)	857 (22.9)	3,079 (34.4)		
30-34	5,843 (46.0)	1,850 (49.3)	3,993 (44.6)		
≥35	2,920 (23.0)	1,043 (27.8)	1,877 (21.0)		
**Ethnic group**				2.217	0.136
Han Chinese ethnic group	12.371 (97.4)	3,641 (97.1)	8,730 (97.6)		
Minority	328 (2.6)	109 (2.9)	219 (2.4)		
**Maternal education**				52.544	**<0.001**
Bachelor degree and above	5,077 (40.0)	1,318 (35.1)	3,759 (42.0)		
High school	3,203 (25.2)	1,005 (26.8)	2,198 (24.6)		
Junior middle school and below	4,419 (34.8)	1,427 (38.1)	2,992 (33.4)		
**Maternal occupation**				26.558	**<0.001**
At home	7,614 (60.0)	2,280 (60.8)	5,334 (59.6)		
Management, professional and technical personnel	1,474 (11.6)	357 (9.5)	1,117 (12.5)		
Business, service, freelance	808 (6.4)	231 (6.2)	577 (6.4)		
Laborer-farmer	888 (7.0)	271 (7.2)	617 (6.9)		
Others	1,915 (15.1)	611 (16.3)	1,304 (14.6)		
**Marriages**				0.758	0.384
Married	12,397 (97.6)	3,654 (97.4)	8,743 (97.7)		
Others	302 (2.4)	96 (2.6)	206 (2.3)		
**Maternal parity (times)**				131.866	**<0.001**
1	7,319 (57.6)	1,890 (50.4)	5,429 (60.7)		
2	3,902 (30.7)	1,286 (34.3)	2,616 (29.2)		
≥3	1,478 (11.6)	574 (15.3)	904 (10.1)		
**Antiviral medication use**				202.885	**<0.001**
No	7,044 (55.5)	2,444 (65.2)	4,600 (51.4)		
Yes	5,655 (44.5)	1,306 (34.8)	4,349 (48.6)		
**Pregnancy duration (weeks)**				34.196	**<0.001**
<37	749 (5.9)	292 (7.8)	457 (5.1)		
≥37	11,950 (94.1)	3,458 (92.2)	8,492 (94.9)		
**Mode of delivery**				2.616	0.106
Vaginal delivery	7,787 (61.3)	2,259 (60.2)	5,528 (61.8)		
Cesarean section	4,912 (38.7)	1,491 (39.8)	3,421 (38.2)		
**Places of delivery**				2.994	0.084
Midwifery institutions at the county level and below	7,591 (59.8)	2,198 (58.6)	5,393 (60.3)		
Municipal and higher midwifery institutions	5,108 (40.2)	1,552 (41.4)	3,556 (39.7)		
**Gender of children**				0.385	0.535
Female	5,787 (45.6)	1,693 (45.1)	4,094 (45.7)		
Male	6,912 (54.4)	2,057 (54.9)	4,855 (54.3)		
**Birth weight of children (g)**				4.123	**0.042**
<2,500	724 (5.7)	238 (6.3)	486 (5.4)		
≥2,500	11,975 (94.3)	3,512 (93.7)	8,463 (94.6)		

Bold values indicate statistical significance (*p* < 0.05).

**Table 5:** Risk factors associated with PVST not being completed of commonly exposed children (*n* = 62,315).

Variables	Total (*n* = 62,315)	PVST completed (*n* = 29,160)	PVST not completed (*n* = 33,155)	χ^2^	*P*
**Maternal age (years)**				1,883.005	**<0.001**
<30	19,220 (30.8)	6,531 (22.4)	12,689 (38.3)		
30-34	27,962 (44.9)	14,333 (49.2)	13,629 (41.1)		
≥35	15,133 (24.2)	8,296 (28.5)	6,837 (20.6)		
**Ethnic group**				0.679	0.410
Han Chinese ethnic group	61,167 (98.2)	28,609 (98.11)	32,558 (98.2)		
Minority	1,148 (1.8)	551 (1.89)	597 (1.8)		
**Maternal education**				466.364	**<0.001**
Bachelor degree and above	27,216 (43.7)	11,402 (39.1)	15,814 (47.7)		
High school	15,345 (24.6)	7,735 (26.5)	7,610 (13.0)		
Junior middle school and below	19,754 (31.7)	10,023 (34.4)	9,731 (29.3)		
**Maternal occupation**				214.658	**<0.001**
At home	32,944 (52.9)	15,244 (52.3)	17,700 (53.4)		
Management, professional and technical personnel	8,665 (13.9)	3,699 (12.7)	4,966 (15.0)		
Business, service, freelance	4,627 (7.4)	1,989 (6.8)	2,638 (8.0)		
Laborer-farmer	4,326 (6.9)	2,250 (7.7)	2,076 (6.3)		
Others	11,753 (18.9)	5,978 (20.5)	5,775 (17.4)		
**Marriages**				5.657	**0.017**
Married	61,198 (98.2)	28,598 (98.1)	32,600 (98.3)		
Others	1,117 (1.8)	562 (2.0)	555 (1.7)		
**Maternal parity (times)**				563.264	**<0.001**
1	34,803 (55.9)	14,827 (50.9)	19,976 (60.3)		
2	19,839 (31.8)	10,238 (35.1)	9,601 (29.0)		
≥3	7,673 (12.3)	4,095 (14.0)	3,578 (10.8)		
**Pregnancy duration (weeks)**				29.540	**<0.001**
<37	3,425 (5.50)	1,757 (6.0)	1,668 (5.0)		
≥37	58,890 (94.5)	27,403 (94.0)	31,487 (95.0)		
**Mode of delivery**				9.161	**0.002**
Vaginal delivery	36,203 (58.1)	16,755 (57.5)	19,448 (58.7)		
Cesarean section	26,112 (41.9)	12,405 (42.5)	13,707 (41.3)		
**Places of delivery**				0.214	0.644
Midwifery institutions at the county level and below	32,795 (52.6)	15,375 (52.7)	17,420 (52.5)		
Municipal and higher midwifery institutions	29,520 (47.4)	13,785 (47.3)	15,735 (47.5)		
**Gender of children**				0.039	0.844
Female	28,382 (45.6)	13,269 (45.5)	15,113 (45.6)		
Male	33,933 (54.5)	15,891 (54.5)	18,042 (54.4)		
**Birth weight of children (g)**				13.322	**<0.001**
<2,500	3,128 (5.0)	1,563 (5.4)	1,565 (4.7)		
≥2,500	59,187 (95.0)	27,597 (94.6)	31,590 (95.3)		

Bold values indicate statistical significance (*p* < 0.05).

### Discussion

WHO and the Centers for Disease Control and Prevention (CDC) in the U.S. have both explicitly recommended PVST for children born to HBsAg-positive mothers (22, 23). This measure is similarly endorsed by the CDC in China within the childhood immunization schedule of the National Immunization Program Vaccines (24). In this regard, Fujian Province published the “*Plan for Implementation of the Action Plan for Elimination of MTCT of HIV/AIDS, Syphilis, and Hepatitis B (2023-2025)*” in 2023, which specifically states that children exposed to Hepatitis B require PVST. The study found that the average PVST coverage of children born with HBV exposure in Fujian Province from 2021 to 2023 was 56.1%, rising from 32.4% in 2021 to 79.2% in 2023;notably, the PVST coverage of children at high risk of HBV exposure increased from 53.8 to 83.5%, indicating a significant improvement; the PVST coverage in 2023 in Fujian Province (79.2%) is higher than that of Zhejiang Province (67.08%) and the Bao' an District of Shenzhen City (56.15%) (25, 26). This could be attributed to the strong coordination across all tiers of the healthcare system in Fujian Province, where primary institutions have established routine phone follow-ups and higher-level hospitals have offered technical advice, greatly enhancing PVST coverage.

Nevertheless, the result shows that most of the children in the study had a gap of over 2 months between their last vaccine and PVST testing. This contrasts with WHO guidance that requires testing between one and 2 months after vaccination (23). The delivery of catch-up vaccines may be delayed as a result of the testing delay, thus increasing the risk of chronic infection and its complications. Consequently, the intervention node should be advanced in the future even if the current follow-up mechanism can raise the testing completion rate but is unable to successfully ensure the timeliness of testing. It is noteworthy that the study achieved an exceptionally high follow-up rate (approximately 100%), substantially surpassing those reported in previous studies (85.77%) (26). The lost-to-follow-up group was characterized by higher proportions of ethnic minorities, lower education, unemployment, multiparity, preterm birth, and highly exposed children. These characteristics suggest that the lost-to-follow-up population may represent a group with distinct socioeconomic and clinical risk profiles (27–29). Therefore, healthcare providers should proactively counsel such high-risk populations about the importance of completing scheduled follow-ups and monitoring pediatric immunization outcomes to minimize potential attrition bias.

The presence of HBeAg and elevated HBV DNA levels are known to be important risk factors for the incidence of MTCT (30–32). Therefore, children who are considered to be at high risk for exposure to HBV require extra attention. The results of this study showed that among children at high risk of HBV exposure, maternal use of antiviral medication during pregnancy (OR = 1.575, 95% *CI* = 1.452–1.708, *P* < 0.01) and pregnancy duration ≥37 (OR = 1.433, 95% *CI* = 1.198–1.714, *P* < 0.01) were protective factors for completing PVST. First, it was noted that pregnant women who finished the entire course of antiviral therapy showed a high level of medical adherence, which may translate to other medical behaviors (such as testing follow-up). Besides, clinicians typically stress to pregnant women the importance of postnatal monitoring and the risk of mother-to-child transmission when they are on antiviral therapy (33, 34). Notably, antiviral medicine is recommended for all HBsAg-positive pregnant women with HBV DNA ≥2 × 10^5^ IU/ml or positive HBeAg to MTCT of HBV (23, 35), however, the data from this study indicate that the proportion of those receiving antiviral medicine (55.5%) still needs to be increased. Additionally, children born at full term who are at high risk of exposure had a greater rate of PVST than children born at less than full term, however, early PVST for infection risk is necessary due to preterm children' immature immune systems (36). According to the systematic monitoring data, follow-up staff should therefore prioritize preterm infants and step up follow-up.

For children with common exposure to HBV, one risk factor for completing PVST was the mother's employment as a laborer or farmer (OR = 0.833, 95% *CI* = 0.780–0.889, *P* < 0.01). Mothers who worked as labors or farmers were less likely than homemakers to have their kids undergo PVST. One possible explanation for this disparity is time or money constraints (37). In light of this, it is advised that PVST testing be included in national or local public health initiatives, offering free or heavily discounted testing services, and that primary care clinics be furnished with the testing apparatus and diagnostic personnel required. Furthermore, employment as a worker or farmer may be indicative of a lower level of health literacy (38, 39).Given that health literacy is a recognized important factor for attending preventive medicine services, parental health education initiatives could be integrated into prenatal care services to enhance parents' health literacy.

There are some risk factors that are present in both common and high HBV viral load groups of mothers. The multivariable analysis revealed that mothers of hepatitis B-exposed children who are aged ≥30 years, have a high school education or less, and have ≥2 deliveries are risk factors for completing PVST. Lower PVST rates were seen in mothers 30 years of age or older, which is in line with the findings of Wang Ronghuan et al (18). On the other hand, in line with Tang Hanyan's findings, the PVST rate rose as the mother's level of education grew (40), which is consistent with Anderson's behavioral model, which holds that people with higher education tend to have more substantial resources and a better understanding of the importance of hepatitis B prevention and control (41, 42). Mothers with more than two children, however, would have a low PVST coverage. This result is in line with the follow-up analysis from Zhejiang (25). Thus, it is recommended that the follow-up mechanism should be optimized in the way of targeting families with mothers aged <30, low maternal education, multiparity or history of non-compliance. Community health workers should initiate advance home visits 2 weeks before the testing window and follow up with regular phone calls until testing is completed.

Some Chinese provinces have faced significant challenges in promoting PVST, including population movement, privacy issues, a lack of public awareness of PVST, and parental objection to the collection of peripheral blood from children [43, 20]. By using the national PMTCT Reporting System to combine disparate data from primary healthcare facilities, disease control centers, and maternal and child health institutions, Fujian Province has developed a strong surveillance framework that allows for real-time data monitoring to identify children who are past due for testing. Although Fujian has made great progress, PVST coverage and timeliness might yet be improved. Future proactive, predictive therapies are suggested as a paradigm change from reactive follow-up. First, we need to incorporate PVST awareness modules into regular prenatal care. Antenatal education classes and mobile health applications should be used to help mothers better understand the dangers of chronic HBV and timely critical monitoring. Then, to highlight the negative effects of postponing testing by providing PVST-focused information during maternal HBV screening and at each of the three kid immunization visits. Additionally, strengthening collaborative, data-driven strategies is essential to sustain progress toward MTCT elimination. It is recommended that the system in the future be able to evaluate historical data to forecast high-risk areas or populations with low PVST rates in order to direct the appropriate allocation of resources. In a word, the transition from “reactive follow-up” to “proactive anticipatory intervention” is expected to enhance coverage in the future while preserving the promptness of immunization surveillance.

## Strengths and limitations

The primary strength of this study lies in its population-based design, utilizing a comprehensive real-world dataset that includes all HBsAg-positive pregnant women in Fujian Province, China. Second, the use of a national-level information direct reporting system guarantees the correctness, timeliness, and homogeneity of data gathering by logically verifying important fields. This study also has several limitations. As a retrospective study, it relies on existing medical records. Unmeasured confounding variables such as economic burden, migration, healthcare access and maternal HBV knowledge may influence PVST completion rates but were not included in the dataset due to the retrospective cohort design. Besides, the data in this study ended on December 31, 2024, which limits the follow-up period for children born in 2023 compared to 2021 and 2022. Moreover, it is possible that some children's follow-up was not fully completed and, as a result, was not included in the study, leading to a possible underestimation of follow-up completion for the last year of the study period.

## Conclusion

This population-based cohort study shows significant progress in PVST coverage and MTCT control among HBV-exposed children in Fujian Province from 2021 to 2023. However, PVST's coverage and timeliness might yet be improved. It is strongly recommended that a standard educational process be integrated into the antepartum and postpartum observation period, with a particular emphasis on low-age pregnant women and those with limited educational attainment or with more than one child.

## Data Availability

The datasets presented in this article are not readily available because the datasets generated and analyzed during this study are not publicly available due to restrictions imposed by Chinese regulations on the protection of personal health information and the management of national disease surveillance systems. The data contain sensitive, identifiable patient information collected through the China Immunization Planning Information System and the China Information System for Prevention of Mother-to-Child Transmission. Access to these data is strictly controlled to ensure patient confidentiality and privacy, in compliance with ethical approvals and relevant data protection laws. Requests to access the datasets should be directed to Xiumin Jiang, jiangxiumin@fjsfy.com.
